# The predictive and prognostic value of weight loss and body composition prior to and during immune checkpoint inhibition in recurrent or metastatic head and neck cancer patients

**DOI:** 10.1002/cam4.5522

**Published:** 2022-12-09

**Authors:** Anna C. H. Willemsen, Nina De Moor, Jeroen Van Dessel, Laura W. J. Baijens, Michel Bila, Esther Hauben, Mari F. C. M. van den Hout, Vincent Vander Poorten, Ann Hoeben, Paul M. Clement, Annemie M. W. J. Schols

**Affiliations:** ^1^ Division of Medical Oncology, Department of Internal Medicine Maastricht University Medical Center+ Maastricht The Netherlands; ^2^ GROW‐School of Oncology and Developmental Biology Maastricht University Medical Center+ Maastricht The Netherlands; ^3^ Department of Respiratory Medicine, NUTRIM School of Nutrition and Translational Research in Metabolism Maastricht University Medical Center+ Maastricht The Netherlands; ^4^ Department of Oncology Leuven Cancer Institute, KU Leuven Leuven Belgium; ^5^ Department of Biomedical Sciences, KU Leuven & Oral and Maxillofacial Surgery University Hospitals Leuven Leuven Belgium; ^6^ Department of Otorhinolaryngology, Head and Neck Surgery Maastricht University Medical Center+ Maastricht The Netherlands; ^7^ Department of Pathology University Hospitals Leuven Leuven Belgium; ^8^ Department of Pathology Maastricht University Medical Center+ Maastricht The Netherlands; ^9^ Otorhinolaryngology Head and Neck Surgery University Hospitals Leuven, Leuven Cancer Institute, KU Leuven Leuven Belgium

**Keywords:** body composition, cachexia, head and neck cancer, immune checkpoint inhibitors, weight loss

## Abstract

**Background:**

Response rates of immune checkpoint inhibitor (ICI) therapy for recurrent and/or metastatic head and neck squamous cell carcinoma (R/M HNSCC) are low.

**Patients and Methods:**

This retrospective multicentre cohort study evaluates the predictive and prognostic value of weight loss and changes in body composition prior and during therapy. Patient, tumor, and treatment characteristics of 98 patients were retrieved, including neutrophil and platelet‐lymphocyte‐ratio (NLR and PLR). Programmed death‐ligand 1 (PD‐L1) expression was determined on residual material. Cachexia was defined according to Fearon et al. (2011). Skeletal muscle (SM), visceral adipose tissue (VAT), and subcutaneous adipose tissue (SAT) were evaluated on computed tomography scans at the third lumbar vertebrae level. Univariable and multivariable regression analyses were performed for 6 months progression free survival (PFS6m) and overall survival (OS).

**Results:**

Significant early weight loss (>2%) during the first 6 weeks of therapy was shown in 34 patients (35%). This patient subgroup had a significantly higher NLR and PLR at baseline. NLR and PLR were inversely correlated with SM and VAT index. Independent predictors of PFS6m were lower World Health Organization performance status (HR 0.16 [0.04–0.54] *p* = 0.003), higher baseline SAT index (HR 1.045 [1.02–1.08] *p* = 0.003), and weight loss <2% (HR 0.85 [0.74–0.98] *p* = 0.03). Baseline cachexia in combination with >2% early weight loss remained a predictor of OS, independent of PD‐L1 expression (HR 2.09 [1.11–3.92] *p* = 0.02, HR 2.18 [1.13–4.21] *p* = 0.02).

**Conclusion:**

We conclude that the combination of cachexia at baseline and weight loss during ICI therapy is associated with worse OS in R/M HNSCC patients, independent of PD‐L1 expression.

## Graphical abstract

The prognostic value of weight loss prior to and during ICI therapy for R/M HNSCC

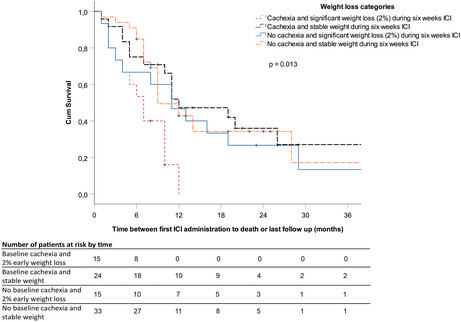

## 1 INTRODUCTION

Immune checkpoint inhibitors (ICI) have become of undeniable value in anti‐tumor treatment, providing successful outcomes in a selection of patients. While ICI therapy is standard of care for first‐line therapy of melanoma and non‐small cell lung carcinoma (NSCLC), ICI therapy for recurrent and/or metastatic head and neck squamous cell carcinoma (R/M HNSCC) is still relatively new. Three pivotal phase III trials concerning programmed death ligand 1 (PD‐L1) targeted immunotherapy in R/M HNSCC have been published.^1^, ^2^, ^3^ An increase in median overall survival (OS) in comparison to standard chemotherapy was found with a durable response. The phase III CheckMate 141 trial resulted in the approval of nivolumab in the second‐line cisplatin refractory R/M HNSCC setting.^3^ Furthermore, the phase III KEYNOTE 040 trial showed similar results with pembrolizumab.^1^ Lastly, the phase III KEYNOTE 048 trial demonstrated the efficacy of pembrolizumab as first‐line treatment for a subgroup of R/M HNSCC patients.^2^ Indeed, unfortunately, only 13%–23% of patients ultimately benefitted from anti‐PD‐1 therapy in these studies, emphasizing the need for better predictive biomarkers to improve patient selection prior to ICI therapy. Patients with tumor cells or tumor infiltrating T‐cells expressing PD‐L1 seem to benefit more from ICI therapy, but PD‐L1 negative tumors are not necessarily ICI‐resistant.^1^, ^2^, ^3^ The combined positive score (CPS, total number of PD‐L1‐positive cells (tumor cells, lymphocytes, and macrophages) divided by the total number of tumor cells multiplied by 100) now serves as a predictive marker of ICI response.^2^ Besides PD‐L1 expression, the number of tumor infiltrating lymphocytes, tumor microenvironment, and tumor mutational burden are being investigated as potential predictive biomarkers.^4^, ^5^ With PD‐L1 CPS as the only predictive biomarker in a standard practice, it remains challenging to identify those patients with low chances of response to avoid unnecessary toxicity and costs without treatment benefit.

Cancer cachexia, a multifactorial syndrome characterized by involuntary weight loss consisting of skeletal muscle and fat mass loss, is a common metabolic problem in HNSCC patients. This is due to the disease itself, to the location of the tumor interfering with adequate caloric intake, and to previous oncological therapy. Cachexia is often accompanied by systemic inflammation, causing a catabolic state that imbalances energy reserves and leads to muscle protein turnover. In turn, this may cause weight loss and muscle mass loss.^6^ This syndrome is associated with higher treatment toxicity and shorter survival.^7^ Whereas the prognostic value of low muscle mass and weight loss during (chemo)radiotherapy in HNSCC has been well established,^8^, ^9^, ^10^ the effects of weight loss and changes in body composition before and during ICI therapy are still underexplored. Studies in lung cancer have presented early weight loss during ICI therapy in terms of visceral and subcutaneous adipose tissue (VAT and SAT) loss and low SM mass at start of the ICI therapy as predictors for OS.^11^, ^12^ A recent study by Arribas et al. has determined the prognostic importance of skeletal muscle mass index (SMI) at baseline in HNSCC patients receiving ICI therapy with or without concurrent chemotherapy.^13^ However, weight loss and changes in body composition prior to and during ICI monotherapy were not studied and adipose tissue compartments were not evaluated separately. Therefore, the aim of this study is to evaluate the predictive and prognostic value of weight loss and changes in body composition prior to and during ICI therapy, considering additional patient, disease, and immune system characteristics. In this context, the effects of weight loss and changes in body composition on six‐month progression free survival (PFS6m), OS, and autoimmune toxicity in R/M HNSCC were explored.

## 2 METHODS

### 2.1 Study design and patient selection

A retrospective study design was completed according to the Strengthening the Reporting of Observational Studies in Epidemiology (STROBE) guidelines.^14^ This study was approved by the Medical Ethics Committee of Maastricht University Medical Center (MUMC+), Maastricht, the Netherlands (METC 2019‐1403), and University Hospitals Leuven (UZL), Leuven, Belgium (S65364). The study sample was derived from a population with R/M HNSCC who received PD‐1 or PD‐L1 inhibitor monotherapy at the department of General Medical Oncology of UZL/Leuven Cancer Institute and the Comprehensive Cancer Center of MUMC+ between January 1st 2014 and March 17th 2020. Patients were excluded if they received concomitant chemotherapy or other immune modulators (e.g., cytotoxic T‐lymphocyte associated protein 4 (CTLA‐4) inhibitors), had a second primary malignancy, had no baseline and/or first follow‐up computed tomography (CT) scan at the level of the third lumbar vertebrae (L3), or if baseline weight measurement was lacking.

Clinical characteristics including patient, tumor, (previous) oncological treatment characteristics, and the amount of previous palliative systemic treatment lines were retrospectively extracted from the electronic health records. At baseline, the World Health Organization performance status (WHO PS)^15^ was determined for every patient by the oncologist. The individual Charlson comorbidity index (CCI)^16^ was calculated based on the medical history reported in the electronic health records. The CCI was dichotomized based on the median. Autoimmune toxicity was evaluated by the oncologist throughout the treatment trajectory using Common Terminology Criteria for Adverse Events (CTCAE).^17^ This variable was dichotomized into CTCAE grade 2 or higher versus CTCAE grade 0 or 1. Based on results from Weber et al.,^18^ the cut‐off for the evaluation period of autoimmune toxicity was set at 6 months after ICI initiation.

Long‐term responders were defined as patients receiving ICI therapy for at least 6 months, in other words, patients who had a progression free survival of more than six months (PFS6m) according to the Response Evaluation Criteria in Solid Tumors (RECIST) 1.1 guidelines.^19^ The six‐month cut‐off was chosen based on a recent meta‐analysis, reporting that 6 month durable response is prognostic of 12 month OS in ICI studies.^20^ OS was evaluated from the first day of ICI administration to the date of death or the date of last follow‐up.

### 2.2 Body composition

Abdominal CT scans performed at baseline and at first evaluation as per internal protocol were collected from the database of the radiology department at UZL and MUMC+, and subsequently pseudonymized. Baseline scans were not older than 30 days at start of ICI therapy. The most cranial CT slice on level L3 clearly displaying both vertebral transverse processes was selected for delineation using sliceOmatic software v5.0 (TomoVision). An experienced researcher (over 750 measured CT scans) delineated the areas of interest on the scans and performed the body composition measurements. The observer was blinded to the moment of CT assessment (baseline vs. follow‐up) and to the identity and medical history of the patients. Cross sectional areas (CSA) of SM, VAT, and SAT were measured using pre‐established thresholds of Hounsfield units (SM −29 to 150, VAT −150 to −50, and SAT −190 to −30). SMI, VAT index (VATI), and SAT index (SATI) were calculated using the CSA of SM, VAT, and SAT each divided by height in meters squared (m^2^).

Low SMI was defined using the cut‐off values for SMI described in 2013 by Martin et al.^21^ Cachexia was defined as weight loss >5% during the past 6 months or body mass index (BMI) <20 kg/m^2^ and weight loss >2% or low SMI and weight loss >2%.^7^ Weight loss during the first 6 weeks of ICI therapy was considered clinically significant in case of 2% or more loss based on the consensus definition of cachexia and the American Society of Clinical Oncology (ASCO) guideline.^7^, ^22^

### 2.3 Inflammatory parameters

Systemic inflammation was evaluated using the inflammatory indices neutrophil‐lymphocyte‐ratio (NLR) and platelet‐lymphocyte‐ratio (PLR).^23^ They were defined as the absolute neutrophil count divided by the absolute lymphocyte count and absolute platelet count divided by the absolute lymphocyte count, respectively, obtained from complete blood count at baseline.

### 2.4 Immunohistochemistry

Representative tumor sections were immunohistochemically stained for PD‐L1 expression using the standardized 22C3 pharmDx assay on the Dako Link 48 platform (Dako). This assay was used as standard in the KEYNOTE‐048.^2^

### 2.5 Pathological assessment of PD‐L1 staining

A dedicated head and neck pathologist, certified for PD‐L1 testing, and an experienced head and neck researcher, assessed stained slides. Any discrepancies were resolved through a consensus discussion. Specimens were scored using CPS. This score was defined as the number of positive tumor cells, lymphocytes, and macrophages, divided by the total number of viable tumor cells multiplied by 100. Clinically relevant cut‐offs of ≥1 and ≥20 for CPS were used. Slides that contained less than 100 viable tumor cells were excluded.

### 2.6 Statistical analysis

Normally distributed variables were reported as means (±standard deviation (SD)). Non‐normally distributed variables were reported as medians (interquartile range (IQR)). Differences between groups were analyzed using independent samples *T*‐test and the Mann–Whitney *U* test, respectively. Categorical variables were analyzed with the Pearson's Chi^2^ test and Fisher's Exact test where appropriate. Correlations were evaluated using Pearson correlation coefficient. The distributions of OS were estimated by the Kaplan–Meier method and compared by means of the log‐rank test. Cox‐proportional hazard models were used to estimate the hazard ratio (HR) and calculate the corresponding 95% confidence intervals (CI's) for OS. Univariable and multivariable binary logistic regression analysis was performed for long‐term response and autoimmune toxicity. Potential predictive and prognostic variables were selected for multivariable analysis using forward stepping analysis with *p* for entry ≤0.10 and *p* to remove upon entry >0.05. Significance was set at the value *p* < 0.05. Changes in CSA of SM, VAT, and SAT were corrected for days between the baseline and follow‐up CT scan in the regression analyses.

All statistical analyses were performed using SPSS (IBM version 25 for Windows). For the Fisher's Exact test with more than two by two items, the online calculator http://vassarstats.net/fisher2×4.html was used.

## 3 RESULTS

Out of the 177 patients treated with ICI, 98 patients met the inclusion criteria for this study. Information on weight change during the 6 months prior to ICI initiation was available for 87 patients. NLR and PLR could be retrieved for 93 patients. PD‐L1 CPS could be determined in 79 patients.

### 3.1 Baseline characteristics of the study population

Baseline characteristics are displayed in Table 1. The population was predominantly male (85%) with a mean age of 63 years and the majority suffered from distant metastatic disease (67%). Forty patients (41%) received ICI therapy as first line palliative treatment. The majority of the patients was treated with nivolumab (61%).

**TABLE 1:** Baseline characteristics

Variable	Total	Stable or increased weight during 6 weeks ICI	At least 2% weight loss 2% during 6 weeks ICI	*p* value
	*N* = 98	*n* = 64	*n* = 34	
Patient characteristics				
Female	15 (15)	7 (11)	8 (24)	0.10^a^
Male	83 (85)	57 (89)	26 (77)	
Age (mean ± SD)	63.2 ± 8.0	63.6 ± 7.9	62.5 ± 8.5	0.52^b^
WHO PS 0	32 (33)	25 (39)	7 (21)	0.14^c^
WHO PS 1	61 (62)	37 (58)	24 (71)	
WHO PS 2	5 (5)	2 (3)	2 (9)	
CCI below 7	35 (36)	20 (31)	15 (44)	0.21^a^
CCI 7 or higher	63 (64)	44 (69)	19 (56)	
Never smoked	6 (6)	5 (8)	1 (3)	0.50^a^
Current smoker	43 (44)	26 (41)	17 (50)	
Former smoker	48 (49)	32 (51)	16 (47)	
Missing	1	1	0	
No alcohol use	4 (5)	1 (2)	3 (10)	0.09^c^
Current alcohol user	50 (64)	35 (71)	15 (52)	
Former alcohol user	24 (31)	13 (27)	11 (38)	
Missing	20	15	5	
Disease characteristics				
Oropharynx	37 (38)	21 (33)	16 (47)	0.48^a^
Hypopharynx	14 (14)	12 (19)	2 (6)	
Oral cavity	23 (23)	15 (23)	8 (24)	
Larynx	12 (12)	9 (14)	3 (9)	
Unknown primary	7 (7)	4 (6)	3 (9)	
Other	5 (5)	3 (5)	2 (6)	
Distant metastatic disease	66 (67)	47 (73)	19 (56)	0.08^a^
Locoregional recurrent disease	32 (33)	17 (27)	15 (44)	
P16+ and/or HPV+ oropharynx	15 (16)	11 (18)	4 (13)	0.47^a^
Other	77 (84)	49 (82)	28 (88)	
Missing	6	4	2	
PDL1 expression				
Low (CPS <1)	22 (28)	16 (31)	6 (21)	0.64^a^
Intermediate (CPS 1–19)	36 (46)	22 (43)	14 (50)	
High (CPS ≥20)	21 (27)	13 (26)	8 (29)	
Missing	19			
Treatment characteristics				
PD‐1 inhibitor	80 (82)	55 (86)	25 (74)	0.13^a^
PD‐L1 inhibitor	18 (18)	9 (14)	9 (27)	
First line palliative systemic therapy	40 (41)	28 (44)	12 (35)	0.42^a^
Second line or higher	58 (59)	36 (56)	22 (65)	
Previous tumor surgery	43 (44)	26 (41)	17 (50)	0.37^a^
No previous tumor surgery	55 (56)	38 (59)	17 (50)	
Previous (chemo)radiation	82 (84)	51 (80)	31 (91)	0.17^c^
No previous (chemo)radiation	16 (16)	13 (20)	3 (9)	
Previous EXTREME regimen	47 (48)	29 (45)	18 (53)	0.47^a^
No previous EXTREME regimen	51 (52)	35 (55)	16 (47)	
Platinum refractory	54 (55)	33 (52)	21 (62)	0.33^a^
Non platinum refractory	44 (45)	31 (48)	13 (38)	
Anti‐tumor therapy in 6 months prior to ICI	60 (61)	37 (58)	23 (68)	0.34^a^
No anti‐tumor therapy in 6 months prior to ICI	38 (39)	27 (42)	11 (32)	
Weight and body composition				
Weight loss in 6 months prior to ICI (%) (median (IQR))	−1.9 (13.2)	−1.9 (12.9)	−3.3 (16.3)	0.63^d^
*n*	87	57	30	
BMI (mean ± SD)	22.2 ± 4.3	22.9 ± 4.5	20.9 ± 3.6	**0.03** ^b^
SMI total (median (IQR))	44.7 (9.6)	45.1 (9.9)	42.2 (10.0)	**0.03** ^d^
VATI total (median (IQR))	23.2 (30.6)	25.4 (40.9)	18.8 (23.2)	**0.02** ^d^
SATI total (median (IQR))	31.4 (33.1)	35.2 (30.4)	24.6 (36.2)	0.13^d^
Low SMI	52 (53)	32 (50)	20 (59)	0.41^a^
Normal SMI	46 (47)	32 (50)	14 (41)	
Cachexia	39 (45)	24 (42)	15 (50)	0.48^a^
No cachexia	48 (55)	33 (58)	15 (50)	
Laboratory findings				
NLR (median (IQR))	4.3 (3.5)	3.7 (2.8)	5.4 (4.5)	**0.008** ^d^
*n*	93	60	33	
PLR (median (IQR))	241.9 (189.8)	217.2 (185.0)	302.7 (167.3)	**0.01** ^d^
*n*	93	60	33	
Albumin (mean ± SD)	39.9 ± 4.3	40.1 ± 4.4	39.5 ± 4.3	0.56^d^
*n*	94	61	33	

*Note*: Patients with at least 2% weight loss during the first 6 weeks of ICI therapy versus patients with stable or increased weight. All variables are considered at baseline (start ICI) unless reported otherwise. Percentages do not always add up to 100% due to rounding off. Bold values denote statistical significance at the *p* < 0.05 level.

Abbreviations: BMI, body mass index; CCI, Charlson Comorbidity Index; CPS, combined positivity score; EXTREME regimen including platinum‐based chemotherapy, 5‐fluorouracil and cetuximab (Vermorken et al. 2008); NLR, neutrophil‐lymphocyte ratio; PLR, platelet‐lymphocyte ratio; SATI, subcutaneous adipose tissue index; SMI, skeletal muscle index; VATI, visceral adipose tissue index; WHO PS, world health organization performance status.

^a^ Pearson Chi‐Square.

^b^ Independent samples *T* test.

^c^ Fisher's Exact Test.

^d^ Mann–Whitney *U* Test.

More than half of the population (53%) had low SMI at start of ICI therapy and 39 out of 87 patients with available data on pre‐treatment weight loss were classified as cachectic (45%).

### 3.2 Early changes in weight and body composition

During the first 6 weeks of ICI therapy, 34 patients (35%) experienced significant weight loss, defined as more than 2% total body weight loss. When compared to patients with stable or increasing weight during ICI therapy, this subgroup presented a significantly higher NLR and PLR at baseline. Additionally, patients with significant weight loss during the first 6 weeks of ICI therapy had a lower BMI at baseline (20.9 ± 3.6 vs. 22.9 ± 4.5 kg/m^2^), which was also reflected in significantly lower SMI and VATI.

**FIGURE 1:**
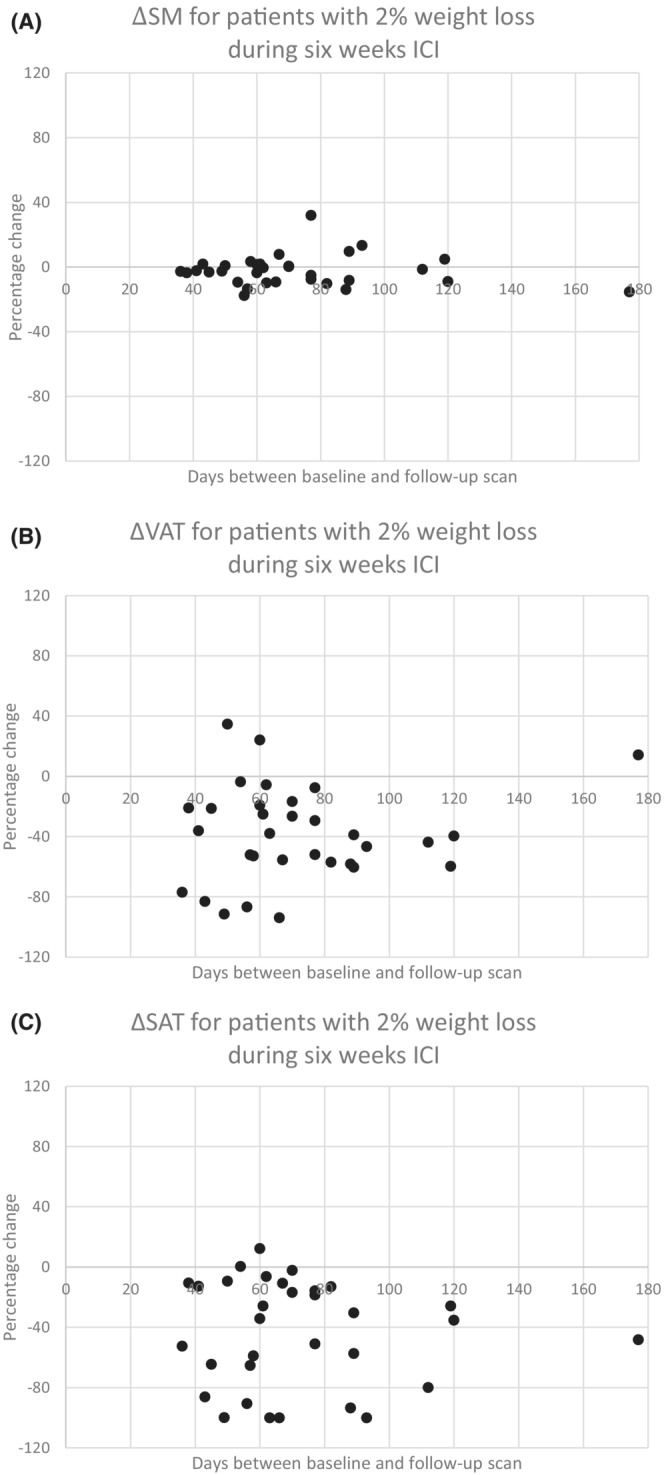
Changes in body composition over time for patients with 2% weight loss during 6 weeks ICI.

To visualize what happened to the specific tissues over time in patients with significant weight loss compared to those with stable or increased weight, the number of days between baseline and follow‐up CT scans were plotted against the percentage change of SM, VAT, and SAT (Figure 1). Patients with early weight loss during 6 weeks of ICI therapy predominantly experienced VAT (1B) and SAT (1C) loss, while loss of SM mass was not distinct (1A).

### 3.3 Systemic inflammation

#### 3.3.1 Neutrophil‐lymphocyte‐ratio

Baseline NLR was not correlated with baseline BMI (*r* = −0.20, *p* = 0.06), but did show a significantly negative correlation with the SMI and VATI (*r* = −0.22, *p* = 0.03 and *r* = −0.27, *p* = 0.009, respectively). No significant correlation was found between NLR and SATI (*r* = −0.17, *p* = 0.12).

#### 3.3.2 Platelet‐lymphocyte‐ratio

Baseline PLR showed a significant correlation with the baseline BMI (*r* = −0.30, *p* = 0.003), reflected in correlations with the SMI (*r* = −0.25, *p* = 0.02) and VATI (*r* = −0.37, *p* < 0.001) but again not significantly correlated with SATI (*r* = −0.20, *p* = 0.06).

#### 3.3.3 Long‐term responders

Thirty‐three patients (34%) continued treatment for 6 months or longer and were considered as long‐term responders in the current study.

Using univariable analysis, the following variables showed potential prognostic value (*p* ≤ 0.10) for long‐term response: lower WHO PS, metastatic disease, PD‐L1 CPS ≥1, higher SMI, higher VATI, higher SATI, and absence of significant weight loss during the first 6 weeks of treatment (Table 2).

**TABLE 2:** Logistic regression analysis for long‐term response defined as more than 6 months progression free survival and ICI therapy continuation

Covariate	*n*	Univariable analysis				Multivariable analysis			
			95% CI				95% CI		
		HR	Lower	Upper	*p* value	HR	Lower	Upper	*p* value
Gender (male)	98	1.48	0.43	5.05	0.53				
Age	98	0.99	0.94	1.04	0.68				
WHO PS 1 or 2 compared to WHO PS 0	98	0.23	0.09	0.57	**0.001**	0.16	0.04	0.54	**0.003**
CCI 7 or higher compared to CCI <7	98	1.78	0.71	4.43	0.22				
Distant metastatic disease versus recurrent only	98	2.32	0.88	6.14	0.09				
P16+/HPV+ oropharyngeal tumors	92	1.47	0.47	4.60	0.51				
PD‐L1 expression									
Low (CPS <1)	22				0.10				
Intermediate (CPS 1–19)	36	4.52	1.13	18.09	**0.03**				
High (CPS ≥20)	21	3.17	0.69	14.46	0.14				
Second line or higher palliative systemic therapy	98	0.75	0.32	1.75	0.51				
Platinum refractory	98	0.94	0.42	2.24	0.94				
Weight loss in 6 months prior to ICI (%, continuous)	87	0.98	0.96	1.01	0.29				
Cachexia and weight loss >2% during 6 weeks ICI (ref)	15				0.14				
Cachexia and stable weight during 6 weeks ICI	24	10.00	1.13	88.91	**0.04**				
No cachexia and weight loss >2% during 6 weeks ICI	15	5.09	0.50	52.29	0.17				
No cachexia and stable weight during 6 weeks ICI	33	10.32	1.21	87.94	**0.03**				
Catabolic category versus others	87	0.11	0.01	0.90	**0.04**				
BMI	98	1.19	1.06	1.34	**0.003**				
SMI	98	1.07	1.01	1.13	**0.02**				
VAT index	98	1.02	1.002	1.04	**0.03**				
SAT index	94	1.03	1.01	1.05	**0.002**	1.05	1.02	1.08	**0.003**
Low SMI	98	0.76	0.33	1.76	0.52				
Cachexia	87	0.66	0.26	1.63	0.36				
Weight loss during first 6 weeks of ICI (%, continuous)	98	0.89	0.80	0.99	**0.03**	0.85	0.74	0.98	**0.03**
NLR	93	0.94	0.80	1.10	0.41				
PLR	93	1.00	1.00	1.00	0.92				
Albumin	94	1.11	0.99	1.24	0.07				

*Note*: All variables are considered at baseline (start ICI) unless reported otherwise. Bold values denote statistical significance at the *p* < 0.05 level.

Abbreviations: BMI, body mass index; catabolic category is defined as the group of patients with cachexia at baseline and further weight loss >2% during 6 weeks immune checkpoint inhibitors; CCI, Charlson Comorbidity Index; ICI, immune checkpoint inhibitors; NLR, neutrophil‐lymphocyte ratio; PLR, platelet‐lymphocyte ratio; SAT, subcutaneous adipose tissue; SMI, skeletal muscle index; VAT, visceral adipose tissue; WHO PS, world health organization performance status.

The WHO PS, SATI, and weight loss during the first 6 weeks of ICI therapy remained significant predictors for long‐term response in multivariable analysis.

### 3.4 Overall survival

At the time of censoring, 69 out of 98 patients (70.4%) had deceased. The median follow‐up was 9 months (range 1–64). At 6 months, OS rate was 72.2% and at 1 year 36.7%.

Using univariable Cox regression analysis, the following variables showed a potential predictive value (*p* ≤ 0.10) for OS: WHO PS, metastatic disease, PD‐L1 CPS ≥1, second line palliative systemic treatment or higher, weight loss during the 6 months prior to ICI initiation, weight loss categories, catabolic category (explanation below), VATI, and weight loss during the first 6 weeks of ICI therapy.

**FIGURE 2:**
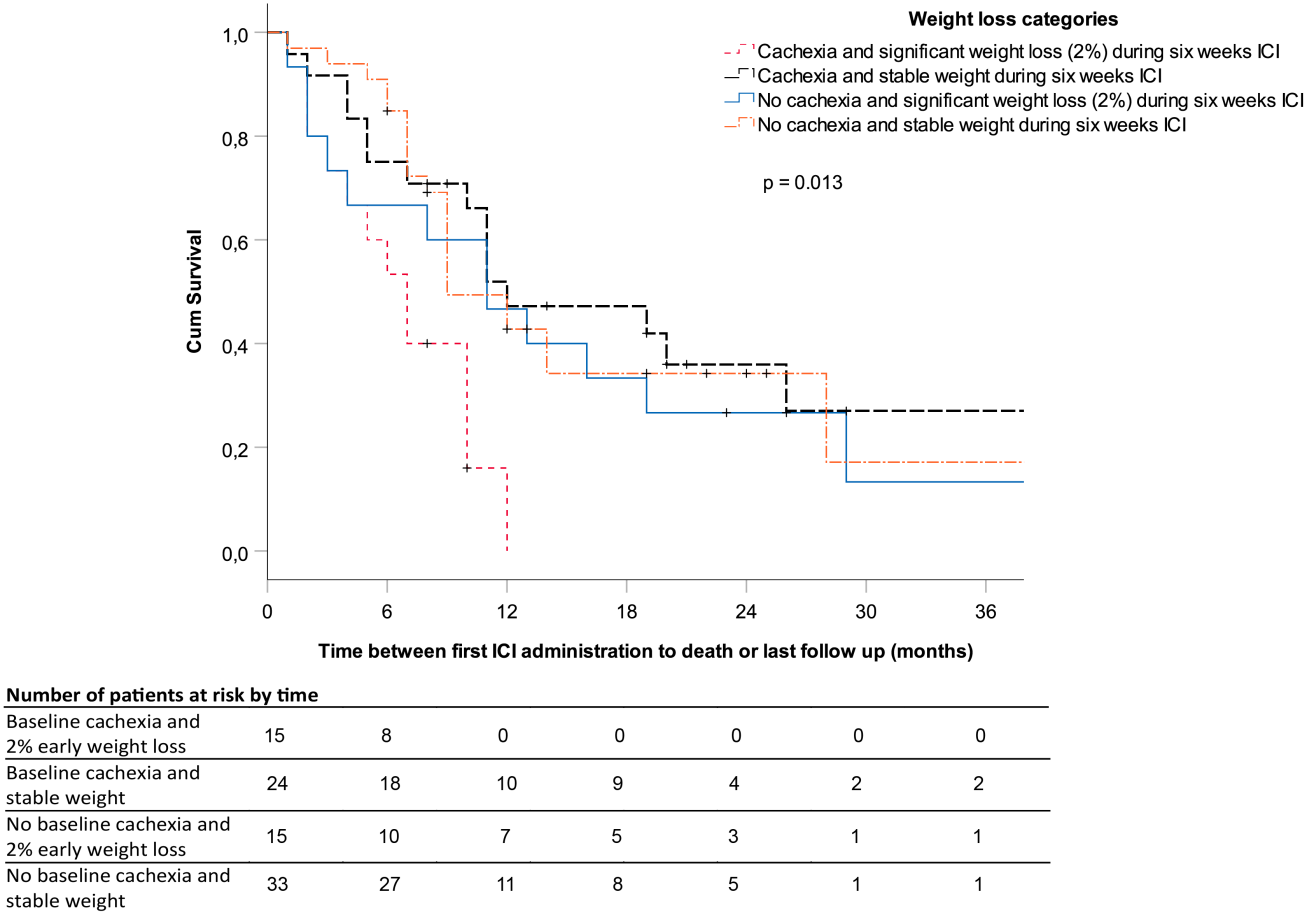
Kaplan Meier survival curve for four categories of cachexia progression.

As weight loss during the 6 months prior to ICI initiation and during the first 6 weeks of ICI therapy were potential predictors for OS, four categories were created to further elucidate the underlying relationships. The cachexia progression categories are as follows: (1) Cachexia at baseline and weight loss >2% during 6 weeks of ICI therapy, *n* = 15, (2) Cachexia at baseline and stable weight during 6 weeks of ICI therapy, *n* = 24, (3) No cachexia and weight loss >2% during 6 weeks of ICI therapy, *n* = 15, and (4) No cachexia and stable weight during 6 weeks of ICI therapy, *n* = 33. The first category was then defined as the catabolic category including patients with progressive weight loss prior and during ICI therapy. The Kaplan–Meier curve for these cachexia progression categories is shown in Figure 2.

In multivariable forward stepwise Cox regression analysis including all the above‐mentioned potential predictors, WHO PS and the catabolic category remained independent significant predictors for OS in the final model (Table 3). When additionally corrected for PD‐L1 expression, only the catabolic category remained a significant predictor.

**TABLE 3:** Cox regression analysis for overall survival

Covariate	*n*	Univariable analysis				Multivariable analysis			
			95% CI				95%CI		
		HR	Lower	Upper	*p* value	HR	Lower	Upper	*p* value
Gender (male)	98	0.73	0.37	1.44	0.36				
Age	98	1.010	0.98	1.04	0.53				
WHO PS 1 or 2 compared to WHO PS 0	98	2.56	1.44	4.55	**0.001**	2.09	1.11	3.92	**0.022**
CCI 7 or higher compared to CCI <7	98	0.76	0.47	1.25	0.28				
Distant metastatic disease versus recurrent only	98	0.66	0.40	1.09	0.10				
P16+/HPV+ oropharyngeal tumors	92	1.08	0.55	2.12	0.82				
PDL1 expression									
Low (CPS <1)	22				0.31				
Intermediate (CPS 1–19)	36	0.62	0.34	1.15	0.13				
High (CPS ≥20)	21	0.74	0.37	1.48	0.39				
Second line or higher palliative systemic therapy	98	1.57	0.94	2.60	0.08				
Platinum refractory	98	1.19	0.74	1.92	0.48				
Weight loss in 6 months prior to ICI (%, continuous)	87	1.02	1.00	1.04	0.06				
Cachexia and weight loss >2% during 6 weeks ICI (ref)					**0.02**				
Cachexia and stable weight during 6 weeks ICI		0.33	0.15	0.71	**0.005**				
No cachexia and weight loss >2% during 6 weeks ICI		0.44	0.19	1.00	**0.05**				
No cachexia and stable weight during 6 weeks ICI		0.38	0.19	0.77	**0.007**				
Catabolic category versus others	87	2.68	1.41	5.11	**0.003**	2.18	1.13	4.21	**0.020**
BMI	98	0.95	0.89	1.02	0.15				
SMI continuous	98	0.98	0.95	1.01	0.21				
VAT index	98	0.99	0.98	1.00	0.10				
SAT index	94	1.00	0.99	1.01	0.86				
Low SMI	98	1.23	0.76	1.97	0.40				
Cachexia	87	1.19	0.72	1.97	0.50				
Weight loss during first 6 weeks of ICI (%, continuous)	98	1.05	1.00	1.10	0.06				
NLR	93	1.04	0.96	1.13	0.38				
PLR	93	1.00	1.00	1.00	0.90				
Albumin	94	0.96	0.91	1.01	0.14				

*Note*: All variables are considered at baseline (start ICI) unless reported otherwise. Bold values denote statistical significance at the *p* < 0.05 level.

Abbreviations: BMI, body mass index; catabolic category is defined as the group of patients with cachexia at baseline and further weight loss >2% during 6 weeks immune checkpoint inhibitors; CCI, Charlson Comorbidity Index; ICI, immune checkpoint inhibitors; NLR, neutrophil‐lymphocyte ratio; PLR, platelet‐lymphocyte ratio; SAT, subcutaneous adipose tissue. SMI, skeletal muscle index; VAT, visceral adipose tissue; WHO PS, world health organization performance status.

To assess which body compartment (SM, VAT, SAT) contributed most to the prognostic value of early weight loss, regression analysis was repeated for change in body composition corrected for days between the baseline and first follow‐up CT scans. In univariable regression analysis, change in VAT was predictive for OS (HR 0.99 [95%CI 0.98–0.99], *p* = 0.009), while SAT change and SM change were not significant (data not shown). VAT change did not remain an independent prognostic factor when entered in multivariable forward stepwise Cox regression including the previously mentioned potential predictors from Table 3.

### 3.5 Immunotherapy induced toxicity

Eighteen patients (18%) experienced autoimmune toxicity CTCAE grade 2 or higher within 6 months after ICI initiation. Autoimmune toxicity included dermatitis (*n* = 6), thyroiditis (*n* = 5), colitis or gastritis (*n* = 3), arthritis (*n* = 2), pneumonitis (*n* = 1), and pericarditis (*n* = 1). Univariable regression analysis to identify potential predictors of autoimmune toxicity revealed a significant predictive value for age with older patients experiencing less immune therapy‐related adverse events (HR 0.92 [0.86–0.99] *p* = 0.02).

## 4 DISCUSSION

This study was conducted to evaluate the predictive and prognostic value of weight loss and changes in body composition prior to and during ICI therapy.

### 4.1 Prognostic and predictive value of weight loss and body composition

In the present population, 45% of the patients were cachectic prior to the start of ICI therapy. The prevalence of cachexia in R/M HNSCC patients has not been described, but our results are comparable to NSCLC patients starting ICI therapy.^24^

Thirty‐five percent of the total population experienced significant weight loss (>2%) during the first 6 weeks of ICI therapy. These patients had a significantly lower BMI at baseline than those with stable or increasing weight. Hypothetically, these patients are in a wasting state that continues during ICI therapy. The low BMI in the subgroup of weight losing patients was reflected in lower values of all three tissue compartments (SMI, VAT, SAT). This subgroup also exhibited a higher NLR and PLR at baseline, as a marker of inflammation. As shown above, patients with baseline cachexia and ongoing significant weight loss during treatment presented with a lower OS. This catabolic category remained a significant prognostic factor, also when corrected for PD‐L1 expression.

Strikingly, baseline SMI was not associated with OS, in contrast with a recent publication by Arribas et al.^13^ In a population of 61 HNSCC patients treated with ICI +/− other agents, including chemotherapy, the authors concluded that a low SMI was associated with worse OS. But weight loss prior to ICI initiation and performance status, both being strong predictors in our study, were not provided. Maybe the dynamic process of weight loss provides more information on ongoing catabolic activity than a potentially stable low muscle mass. The OS results of the present study are comparable to a Japanese retrospective analysis of 42 R/M HNSCC patients treated with nivolumab.^34^ Ueki et al. reported an independent prognostic value of WHO PS as well and of the modified Glasgow prognostic score. The latter emphasizes the role for systemic inflammation, as this score includes a combination of C‐reactive protein and albumin levels. NLR and PLR were used as inflammatory markers in our study and showed an inverse correlation with body composition parameters. Additionally, body weight loss >5% over the 6 months prior to ICI therapy showed a trend toward worse OS in Ueki et al.'s univariable analysis.^34^

Patients presenting with cachexia at baseline but stable weight during ICI therapy, indicating an arrest in catabolism, showed significantly better survival outcomes. Although baseline cachexia may not predict treatment outcome, the evolution of body weight appears a relevant parameter. Only few studies have reported on ‘early’ changes in body composition during the first weeks of ICI therapy, none in HNSCC patients.^12^, ^26^ Crombé et al. performed a retrospective study in patients with metastatic solid tumors treated with ICI therapy, no HNSCC patients were included. The authors reported that baseline body composition parameters did not affect the PFS, while decrease in the psoas muscle index and SATI during the first weeks of treatment were predictive for worse PFS. In addition, low fat mass after ICI initiation contributed to a higher risk of disease progression. OS analysis was not performed.^26^ Previous research in NSCLC patients treated with nivolumab has shown that weight loss, characterized by loss of VAT and SAT at week 6 of treatment, is a significant prognostic factor for poor OS in patients with stage IV NSCLC.^12^

Long term responders, with treatment duration of more than 6 months, made up one third of our study population (34%). Reported PFS6m was only 19.7% in the Checkmate 141 study,^27^ 25.6% in KEYNOTE‐040,^1^ and 25% in the KEYNOTE‐048 (arm with pembrolizumab monotherapy).^2^ This discrepancy might be due to continued treatment based on observed clinical benefit. Patients with stabilizing or ameliorating symptoms might have continued ICI therapy despite CT graphic progression, mislabeling some with true progression as pseudo‐progression. A secondary analysis of KEYNOTE‐048 trial showed a shorter PFS in patients with an intermediate CPS and a trend for better PFS in the CPS ≥20 subgroup when comparing pembrolizumab monotherapy versus chemotherapy.^28^ However PD‐L1 CPS ≥1 was no predictor of PFS6m after multivariate analysis in our population. Differences in CPS distribution might explain this result (CPS 1–19 46% and CPS ≥20 27% in our population versus CPS 1–19 41% and CPS ≥20 44% in KEYNOTE‐048). As PFS seemed a challenging outcome measure due to the concept of pseudo‐progression,^29^ formal PFS analysis was not performed. Instead, OS was used as the primary outcome measure.^30^

When focusing on OS, PD‐L1 CPS was not a prognostic factor in this study population as opposed to previous data.^2^, ^31^ In clinical practice, PD‐L1 CPS is used as criteria for reimbursement and a predictive biomarker.^32^, ^33^ Different from KEYNOTE‐048, we included a heterogeneous population with recurrent and metastatic disease, including patients who already received multiple treatment lines.

### 4.2 Major contribution of adipose tissue

Overall, weight loss during ICI therapy remains of prognostic value, more than just baseline cachexia. This weight loss seems to consist of mainly fat mass loss, both VAT and SAT. SAT was found to be an important indicator of clinical outcomes in the current study cohort, which is consistent with the findings of Martini et al.^35^ A study in 55 nivolumab‐treated NSCLC patients showed that low subcutaneous fat mass was significantly associated with poor overall survival.^36^ These results support the hypothesis that preservation of fat tissue might play a bigger role in ICI therapy compared to chemotherapy.

Studies on body composition in cancer patients receiving chemotherapy mainly showed a reduction of muscle mass and function during treatment. The findings concern head and neck cancer, lung cancer as well as other cancer sites.^37^, ^38^, ^39^, ^40^ The catabolic effects of chemotherapy probably play a major role here. For example, cisplatin is known to activate nuclear factor kappa‐B cells (NFκB), a key player in inflammation and a trigger for muscle wasting.^25^ For immunotherapy, an interaction between ICI and adipose tissue is considered plausible. Adipose tissue is considered an important endocrine organ. It regulates the immune system and the patient's metabolism through circulating adipokines, as observed in obesity.^41^ PD‐L1 expression on adipocytes increases during adipogenesis,^42^ which suggests that a higher fat mass may promote tumor immune evasion. ICI therapy causes increased effector T‐cell activity. As such, preservation of adipose tissue may lead to a more robust host immune response to immunotherapy.^35^

### 4.3 Limitations

The results need to be considered in the light of a number of limitations. First, accurate body composition evaluation requires CT scans at the level of L3 and therefore patients without baseline and follow‐up CT abdomen were excluded. This could have led to a higher percentage of patients with metastatic disease in the study sample, as these patients received extended CT scans instead of a CT scan of the head and neck region only. Patients with distant metastatic disease receiving ICI therapy had better response in the KEYNOTE‐048 study compared to patients with locoregional recurrence only.^2^, ^43^ Even so, 67% of our population had metastatic disease compared to 72% in KEYNOYE‐048 and 47% in CHECKMATE‐141.^1^, ^2^, ^27^ Hence, despite our exclusion criteria, recurrent disease was adequately represented in this real life data set.

Because of the multi‐center study setting, CT scan‐protocols may have differed in slice thickness and dose. Nevertheless, standardized reference points were used for L3 slide selection, and one experienced researcher delineated the structures.

Furthermore, the TNM‐classification changed from the seventh to the eighth edition during the study period. In our dataset both the seventh and eighth editions have been used for staging. However, the definition of metastatic HNSCC did not change and tumor stages at the initial diagnosis were not included in the present analysis.

Lastly, a trend was observed toward more patients with locoregional recurrent disease experiencing significant weight loss compared to patients with distant metastatic disease. In HNSCC, weight maintenance is particularly challenging due to tumor and previous treatment induced symptoms such as xerostomia, oropharyngeal dysphagia, or odynophagia. The contribution of these factors to weight loss could not be evaluated in this study sample. Retrospective analysis of nutritional interventions was considered unreliable and therefore not included in the analysis.

### 4.4 Clinical implications

Assessing tumor response is not always clear‐cut based on radiological criteria alone, especially at the first evaluation during ICI therapy. A better understanding of the relationship between a patient's metabolic state and ICI response will help to select patients more accurately and improve the efficacy of ICI treatment in the R/M setting. As such, tracking of weight changes and body composition may prove valuable in the early decision making regarding (dis)continuation of ICI therapy.

## 5 CONCLUSION

The combination of cachexia at baseline and ongoing weight loss during ICI therapy is associated with worse OS in R/M HNSCC patients, independent of PD‐L1 expression, and is predominantly reflected in loss of fat mass. Reversal of weight loss during ICI therapy predicts significant better OS. The underlying mechanisms of continuous weight loss remain unclear. Additional research is needed to define liquid or tumor‐related (inflammatory) biomarkers, identifying the catabolic patient subgroup and additionally pave the way toward improving ICI efficacy.

## AUTHOR CONTRIBUTIONS

**Anna C. H. Willemsen:** Data curation (equal); formal analysis (equal); investigation (equal); methodology (equal); project administration (equal); validation (equal); visualization (equal); writing – original draft (equal). **Nina De Moor:** Data curation (equal); formal analysis (equal); investigation (equal); methodology (equal); project administration (equal); validation (equal); visualization (equal); writing – original draft (equal). **Jeroen Van Dessel:** Investigation (equal); resources (equal); writing – review and editing (equal). **Laura W. J. Baijens:** Supervision (equal); writing – review and editing (equal). **Michel Bila:** Writing – review and editing (equal). **Esther Hauben:** Investigation (equal); resources (equal); writing – review and editing (equal). **Mari F. C. M. Van den Hout:** Investigation (equal); resources (equal); writing – review and editing (equal). **Vincent Vander Poorten:** Resources (equal); writing – review and editing (equal). **Ann Hoeben:** Conceptualization (equal); methodology (equal); resources (equal); supervision (equal); writing – review and editing (equal). **Paul M. Clement:** Conceptualization (equal); resources (equal); supervision (equal); writing – review and editing (equal). **Annemie M. W. J. Schols:** Conceptualization (equal); methodology (equal); supervision (equal); writing – review and editing (equal).

## CONFLICT OF INTEREST

A.C.H.W., N.D.M., J.V.D., M.B., L.W.J.B., E.H., M.V.D.H., V.V.P., A.H., and A.M.W.J.S. declare that they have no known competing financial interests or personal relationships that could have appeared to influence the work reported in this paper. P.M.C. declares the following financial interests which may be considered as potential competing interests: study budget funds from AstraZeneca; was advisory board member for AbbVie, AstraZeneca, Bayer, Bristol Myers Squibb, Daiichi‐Sankyo, Leo Pharma, Merck Serono, MSD, and Vifor Pharma.

## Data Availability

The data that support the findings of this study are available from the corresponding author upon reasonable request.
